# Bioavailability of Functional Iron in Protein Microparticles

**DOI:** 10.3390/nu18071102

**Published:** 2026-03-30

**Authors:** Saranya Chaiwaree, Radostina Georgieva, Till Deckart, Juliane Lenz, Thawanrat Choonukoolphong, Sureeporn Suriyaprom, Ausanai Prapan, Nitsanat Cheepchirasuk, Axel Pruß, Yu Xiong, Yingmanee Tragoolpua, Hans Bäumler

**Affiliations:** 1 Institute of Transfusion Medicine, Charité-Universitätsmedizin Berlin, 10117 Berlin, Germany; saranya_c@payap.ac.th (S.C.); radostina.georgieva@charite.de (R.G.); till.deckart@outlook.de (T.D.); juliane.lenz.office@web.de (J.L.); thawanrat.choo@gmail.com (T.C.); ausanaip@nu.ac.th (A.P.); nitsanat_cheep@cmu.ac.th (N.C.); axel.pruss@charite.de (A.P.); yu.xiong@charite.de (Y.X.); 2 Department of Pharmaceutical Technology and Biotechnology, Faculty of Pharmacy, Payap University, Chiang Mai 50210, Thailand; 3 Department of Medical Physics, Biophysics and Radiology, Medical Faculty, Trakia University, 6000 Stara Zagora, Bulgaria; 4 Department of Biology, Faculty of Science, Chiang Mai University, Chiang Mai 50200, Thailand; suriyaprom.sureeporn@gmail.com (S.S.); yboony150@gmail.com (Y.T.); 5 Department of Radiological Technology, Faculty of Allied Health Sciences, Naresuan University, Phitsanulok 65000, Thailand

**Keywords:** Caco-2 cell, iron bioavailability, protein microparticles

## Abstract

Background: Iron deficiency remains a major nutritional challenge, partly due to the limited stability and bioavailability of conventional iron formulations in foods and during digestion. In this study, iron–protein microparticles (IP-MPs) based on bovine serum albumin (IA-MPs) and hemp protein (IH-MPs) were developed via coprecipitation and evaluated as food-compatible iron delivery systems. Methods: Iron–protein microparticles (IP-MPs) were fabricated by a coprecipitation technique. The stability of IP-MPs was investigated in a three-phase digestion model. The uptake of IP-MPs by Caco-2 cells as well as the Ferritin concentration in Caco-2 cells were investigated. Results: Particle morphology and size distribution were strongly dependent on the protein matrix, with hemp protein microparticles exhibiting greater size uniformity and higher stability under simulated gastric conditions. In a standardized in vitro gastrointestinal digestion model, both IP-MP formulations preserved iron predominantly in the bioactive Fe(II) state and remained sufficiently intact to reach the intestinal phase. Biocompatibility and iron uptake were assessed using Caco-2 cell monolayers. Neither formulation induced cytotoxic effects, while iron delivered via IP-MPs showed enhanced cellular uptake compared to a commercial iron supplement and ferrous sulfate. The amount of Fe(II) detected in the basolateral compartment of IH-MP and IA-MP samples (1.4 µg and 1.3 µg, respectively) was higher than that observed for Floradix^®^ samples (approximately 0.7 µg) and corresponded to about 25% of the total iron applied. Functional iron bioavailability, assessed by ferritin formation, was significantly higher for IP-MPs, with hemp protein microparticles yielding the strongest ferritin response. Conclusions: These results demonstrate that iron–protein microparticles, particularly those based on hemp protein, effectively improve iron stability during digestion and enhance cellular iron bioavailability, highlighting their potential for application in iron fortification and functional food systems.

## 1. Introduction

Iron is an essential micronutrient vital for numerous physiological processes in the human body, including oxygen transport, DNA synthesis, and cellular respiration. Despite its abundance in nature and the human diet, iron deficiency is considered the most common nutrient deficiency worldwide and affects around two billion people, mainly in developing countries where malnutrition is prevalent, especially young children, people with increased iron needs, etc. [1,2,3]. It affects women with heavy menstrual bleeding [4] and people with restricted or strict dietary habits (e.g., vegans) [5]. The number of vegans and vegetarians has increased steadily in recent years and is expected to continue to rise in the coming years as more and more people recognize the benefits of a vegetarian lifestyle for the environment and their own health [6]. In addition, regular blood donors empty their iron stores, which can only be replenished to a limited extent if the diet is inadequate [7,8].

A key factor contributing to widespread iron deficiency is the low bioavailability of iron from food, which refers to the ratio of ingested iron to that absorbed and utilized by the body. Dietary iron absorption is influenced by both the physicochemical form of iron and nutritional factors that affect its absorption in the gastrointestinal tract.

**Figure 1:**
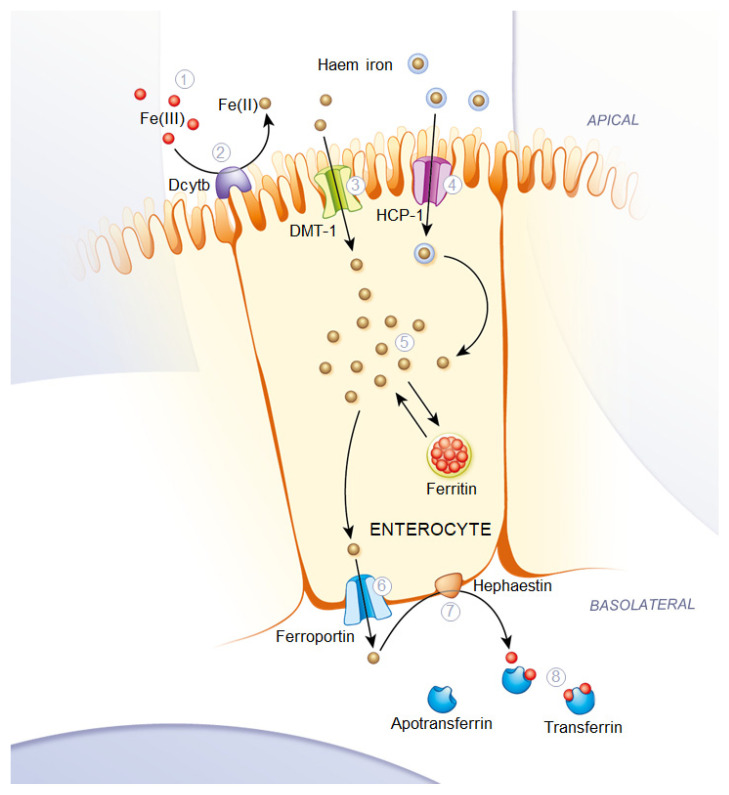
Schematic representation of Fe(II) uptake by intestinal epithelial cells through transport via DMT-1 (3) and HCP-1 (4), intracellular storage as ferritin and the basolateral transport of divalent iron through ferroportin (6) with subsequent oxidation by hephaestin (7), and binding to apotransferrin (8) with resulting removal of the transferrin via the blood route; image from [12].

Iron occurs in food in two different forms: heme iron and non-heme iron. Heme iron, which originates from hemoglobin and myoglobin, is found mainly in animal-based foods, while non-heme iron is present in both plant and animal-based foods. The ratio of these two forms determines the bioavailability of iron in food. Although 10–15% of total dietary iron originates from heme iron in meat-containing diets, it contributes to about 40% of the daily iron requirement because of its higher absorption of 25% [9,10,11]. Heme iron is in the ferrous state Fe(II) and can be absorbed across the duodenal enterocyte brush border membrane though the HCP-1 channel. Additionally, the DMT-1 channel performs the transportation of free ferrous iron into the enterocytes. In the cytoplasm of these cells Fe(II) is either stored as ferritin or exported via ferroportin on the basolateral membrane. Exported Fe(II) is then converted to Fe(III) by the ferroxidase hephaestin to facilitate its binding with plasma transferrin in the blood and get circulated in the body (Figure 1).

In contrast to the heme iron, plant sources contain iron in the ferric state Fe(III) with low solubility, which needs to be converted to the ferrous state (Fe^2+^). Therefore, the bioavailability is low [13].

In addition, several dietary and physiological factors can influence the bioavailability of iron [14]. Vitamin C can reduce Fe(III) to Fe(II), and thus indirectly increase the absorption of iron [15,16,17]. Phytates are found in whole-grain products, legumes, and seeds. They bind iron (Fe^3+^) and form insoluble complexes, thereby reducing its bioavailability [18]. This holds also for polyphenols present in tea, coffee, and certain vegetables [19,20,21]; high levels of Calcium [22,23,24] as well as certain gastrointestinal disorders, such as celiac disease and inflammatory bowel disease, can impair iron absorption [25].

Iron salts, sulphate, fumarate and gluconate are considered standard preparations for the therapy of iron-deficiency anemia (IDA) [26,27]. However, due to gastrointestinal disturbances frequently associated with the use of these preparations, their actual efficacy may be limited [28]. Therefore, the development of an iron preparation with high bioavailability that minimizes the known side effects remains a challenge. Heme iron (blood peptonates) has a high bioavailability [29]. The European Food Safety Authority Panel on Food Additives and Nutrient Sources added to Food declared, due to epidemiological and animal studies, that a high intake of heme iron may be associated with an increased risk of colon cancer [30]. Heme iron (blood peptonates) was not recommended as food supplement.

In the last 10–20 years, public interest in plant-based foods has increased dramatically driven by concerns about climate and animal welfare. This is reflected in the growing number of plant-based foods available. The search for suitable plant-based sources with high protein contents is particularly noteworthy. This has led to a continuous stream of new products on the market, including, more recently, hemp protein powder, produced from the seeds of *Cannabis sativa* L., a cannabis variety that contains little to no Δ6-tetrahydrocannabinol (THC) [31]. Analysis of the protein composition of hemp flour revealed approximately 65% edestin, with the remainder consisting mainly of albumin [32,33,34]. These two proteins have already been shown to have the ability to scavenge free radicals [33,34,35]. Thus, hemp protein, through its antioxidant properties, also could contribute to the protection of iron from oxidation.

Therefore, we fabricated iron–protein hybrid microparticles (IP-MPs) using bovine (animal) albumin (IA-MPs) and hemp (plant) proteins (IH-MPs) in a co-precipitation procedure [36]. The anti-oxidative properties of these proteins should protect the ferrous iron against oxidation. The addition of protein is another positive component for a dietary supplement. This iron formulation should provide more stability in the stomach and therefore a higher bioaccessibility, defined as the quantity of iron that is released from the food matrix in the gastrointestinal tract, which influences the bioavailability being available for physiological functions. Another reason why we also used bovine serum albumin is that we have already used this system for various drug delivery systems [37], so the IA-MPs could serve as a reference for us.

The aim of the study was to determine whether the IP-MPs can reach the small intestine in an in vitro digestive model. To test the bioavailability of iron in the IP-MPs, the Caco-2 cell monolayer model of the intestinal epithelium was established. The bioavailability was evaluated through the uptake of IP-MPs by the Caco-2 cells and the production of intracellular ferritin.

## 2. Materials and Methods

The listed materials were used for the investigations: albumin from bovine serum, fraction V from Sigma Aldrich (Steinheim, Germany); Bradford reagent, calcium chloride, disodium 4-[3-pyridin-2-yl-6-(4-sulfonatophenyl)-1,2,4-triazine-5-yl] benzosulfonate, acetic acid, ethanol, L-ascorbic acid, sodium carbonate, and bovine bile acid (Sigma Aldrich Corporation, St. Louis, MO, USA); ammonium carbonate, magnesium chloride hexahydrate, sodium hydroxide, pepsin from pig gastric mucosa, buffer solution (of pH 4.00, 7.00, and 9.00 ± 0.02 (20 °C)), ROTI^®^CALIPURE, and trichloroacetic acid (Carl Roth GmbH & Co. KG, Karlsruhe, Germany); Dulbecco’s phosphate-buffered saline (DPBS) from PAN-Biotech GmbH (Aidenbach, Germany); Pierce™ BCA Protein Assay Kit and hydrochloric acid (Thermo Fisher Scientific, Waltham, MA, USA); Pancreatin from porcine pancreas, sodium hydrogen carbonate, and potassium hydrogen phosphate (Merck KGaA, Darmstadt, Germany); Pefabloc^®^ SC and AEBSF (Roche Diagnostic GmbH, Berlin, Germany); Iron (II) sulfate heptahydrate (Honeywell Fluka™, Morristown, NJ, USA); potassium chloride and sodium chloride (AppliChem GmbH, Darmstadt, Germany); potassium acetate (Alfa Aesar, Ward-Hill, MA, USA); Human Ferritin ELISA Kit (Thermo Scientific, Frederick, MD, USA); and Cell counting Kit CCK-8 (Dojindo EU GmbH, Munich, Germany).

### 2.1. Purification of Hemp Protein

Hemp protein was extracted from hemp protein powder (Biophyll, Dietersburg, Germany) using the alkaline extraction technique, which uses an alkaline solution to break the bonds within plant cells, allowing the proteins inside to leave the cells. Then, Ultra-turrax was used to break the cells again and increase the extraction efficiency. It was then heated to accelerate the breakdown of the protein structure and centrifuged at 16,000× *g* for 15 min to separate the protein from cell debris. To further purify the protein, it was filtered with a 0.22 µm membrane filter. Finally, the protein concentration was determined using the BCA method.

### 2.2. Fabrication of Iron Protein Microparticles

Co-Precipitation to Produce Iron–Protein Microparticles: A technically simple precipitation process was performed to produce IP-MPs. Two 0.25 M equimolar solutions of Na_2_CO_3_ and FeSO_4_∙7H_2_O were used. The protein (5 mg/mL) was dissolved in the sodium carbonate solution first and then the two solutions were mixed for 30 s to obtain the iron–protein hybrid microparticles (FeCO_3_ with entrapped protein). According to Directive 2002/46/EC of the European Parliament and of the Council, iron carbonate is an approved mineral compound that may be used as a dietary supplement or to fortify food in Europe [38].

### 2.3. Size and Zetapotential

The size of the IP-MPs was measured by means of a Zetasizer (Nano ZS, Malvern, UK). To measure the size distribution of the IP-MP suspension, a 1.5 mL sample of the well-mixed suspension was taken and diluted 1:14 in 0.9% NaCl (*w*/*v*) and three runs with 12 measuring points each were measured. The zeta potential was calculated based on the measured electrophoretic mobility of the particles according to the Smoluchowski equation [39], which, however, only applies to naked, spherical particles to a limited extent. The particle suspension was diluted with DI water to always achieve the same particle concentration and electrical conductivity.

### 2.4. Determination of Iron Concentration

Fe (II) concentration in the various samples was determined using the ferrozine test (disodium 4-[3-pyridin-2-yl-6-(4-sulfonatophenyl)-1,2,4-triazin-5-yl] benzosulfonate) [40,41,42]. The ferrozine method is based on complex formation of ferrozine and Fe(II) ions—a ferrozine ligand coordinates with two nitrogen atoms on Fe^2+^ ions in an aqueous solution. This creates a magenta-colored [Fe(ferrozine)_3_]_4_ complex, whose absorption (maximum at 562 nm) was used to determine Fe(II) concentrations. To determine the total iron concentration, any Fe(III) present must be reduced to Fe(II) with 1 M ascorbic acid before the Ferrozine assay can be used. The iron concentration determinations were carried out both in the supernatant (after centrifugation at 10,000× *g* for 10 min) and in the sediment. After decanting the supernatant, the sediment was brought to the original volume of 2 mL with 2 M HCl so that the IP-MPs dissolved and total iron in dissolved form could be determined using the ferrozine assay.

### 2.5. Determination of Protein Concentration

The protein concentration was determined on the one hand with the Bradford assay [43,44] and on the other hand with the bicinchoninic acid assay (BCA) [45]. Limitations of the Bradford Protein Assay are that certain detergents and components from biological samples interfere with the Bradford assay. Compared to the Bradford assay, the BCA test is particularly characterized by the fact that interference caused by different protein compositions occurs less frequently and the method is more compatible with detergents. However, the BCA is more expensive, more complex, and requires a longer incubation time than the Bradford assay. Particularly noteworthy are the high sensitivity and the large quantifiable concentration range [46]. The protein concentration was measured both in the supernatant after centrifugation for 10 min at 10,000× *g* (Bradford assay) and in the sediment after its dissolution with EDTA (BCA). Since the EDTA used interferes with the color reaction of the BCA and is not compatible with the assay in the concentration required for dissolution, it must be removed by ultracentrifugation [47].

In vitro studies, animal bioassays and human trials are used to estimate iron bioavailability. Human and animal studies of iron absorption and availability are time-consuming and expensive. Therefore, in vitro digestion combined with Caco-2 cell monolayers were applied [48,49,50,51].

### 2.6. Digestion Model

**Figure 2:**
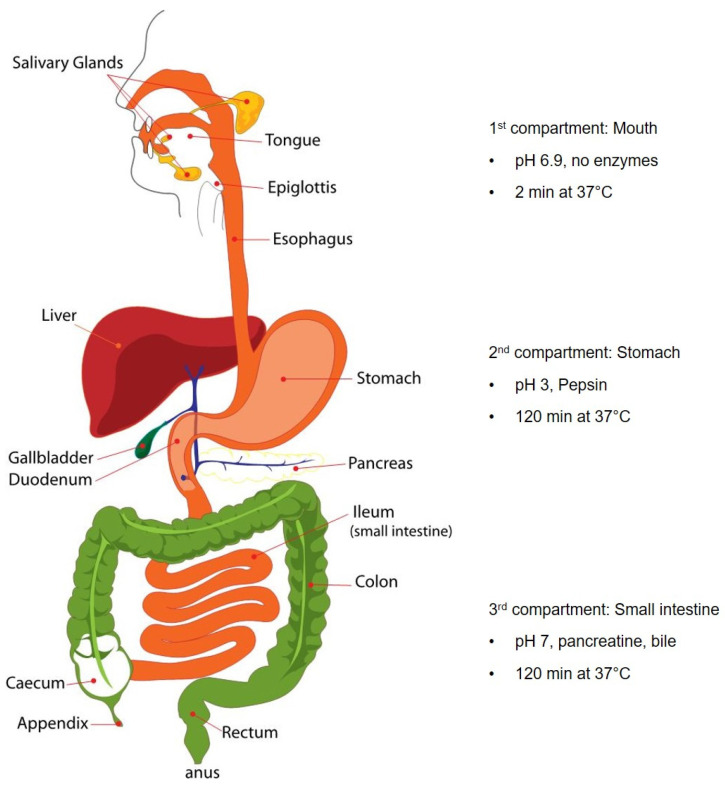
The three-phase static digestion model, modified according to [54].

Experimental procedure of the digestion model (Figure 2). Several implemented protocols exist, with widely accepted results among regulatory institutions. We chose a standardized three-phase static digestion model for the in vitro investigation of the digestion of IP-MPs [49,52,53]. The model includes three compartments of the digestive tract (mouth, stomach, and intestines), in which a physiological environment is modeled in vitro (Figure 2). This model allows for the in vitro investigation of the stability of IP-MPs during the digestion and to analyze the state of iron as well as of IP-MPs in each phase separately and after each step of digestion.

After synthesis, 10 mL of the IP-MP suspension was removed for digestion in the 1st compartment (mouth—oral phase—OrP) and mixed with 8 mL of simulated salivary fluid (SSF) stock solution (for composition, see Supplementary Materials) while stirring at 200 rpm on a magnetic stirrer. An additional 50 µL of CaCl_2_ and 1.95 mL of DI water were added, and the mixture was stirred for 2 min at 200 rpm (pH 6.9). Calcium chloride was added to the oral solution as an enzyme cofactor for amylase, initiating the breakdown of carbohydrates in food and as saliva simulation. A 2 mL aliquot was taken to determine the Fe(II) and total Fe concentration of the supernatant or sediment, and the measurements were carried out as described above. A 1.5 mL aliquot was used for microscopy, as well as DLS measurements and particle volume concentration measurements. All measurements were carried out directly to provide the most accurate representation of the particle suspension at the time of the sample. The samples for determining the Fe concentrations were reduced to determine the initial concentration or diluted to an acidic pH value so that they can be stored in a chemically stable manner for several hours and then measured. Only the samples for measuring the protein concentration were not measured over the experimental duration of the digestion model but were shock-frozen and measured at a later point in time. An amount of 15 mL of the OrP was transferred to a new beaker for use in the second compartment of the digestion model, the gastric phase (GaP).

The first proteolytic digestion of the IP-MPs took place in the GaP. In the GaP, 15 mL of the OrP was mixed with 14.25 mL of the simulated gastric fluid (SGF) (composition see Supplementary Materials) containing pepsin (activity of 2000 units/mL) in a beaker while stirring at 200 rpm. Additionally, 7.5 µL CaCl_2_, 82.5 mL DI water, and, to adjust the pH value to pH 3, 280 µL 2 M HCl and 360 µL 6 M HCl were used. Of the 30 mL volume, 4 reaction vessels (RVs) were filled with 2 mL each during the GaP. The rest of the suspension was filled into a 50 mL RV. All RVs were clamped in an overhead mixer at 25 rpm and shaken at 37 °C in an incubator for 120 min. After 30/60/90/120 min of incubation, an aliquot was removed, and the same measurements were carried out as described above. There were experimental changes in the preparation of the sample for protein concentration measurement. Centrifugation of the sample was performed at 4 °C instead of RT to slow down the ongoing enzymatic reaction. To store the samples, 84 mg of sodium bicarbonate was added to the volume of the sample supernatant (200 µL) to prevent further proteolytic degradation by pepsin. The samples were then shock-frozen at −80 °C to stop the degradation reaction as abruptly as possible. After 120 min of incubation, 20 mL of the GaP volume was transferred to a beaker and used for the InP (intestinal phase of the model).

To the 20 mL of the GaP particle suspension, 11 mL of SIF (simulated intestinal fluid) (for composition, see Supplementary Materials) with 11.43 g of pancreatin of known activity (stored on ice) and 1.305 g of bile acid in 2.5 mL of SIF (freshly prepared) were added. Amounts of 40 µL 0.3 M CaCl_2_ and 1.46 mL 1 M NaOH were also added while stirring at 200 rpm on a magnetic stirrer so that a pH value of 7 was achieved. Four 2 mL aliquots of the 40 mL suspension were filled into RVs and the rest of the suspension was transferred into a 50 mL RV. All RVs were then clamped in an overhead mixer at 25 rpm and incubated in an incubator at 37 °C for 120 min.

After 30/60/90/120 min of incubation, an aliquot was removed, and the same measurements were carried out as described above. There were experimental changes in the preparation of the sample for protein concentration measurement. Centrifugation of the sample was performed at 4 °C instead of RT to slow down the ongoing enzymatic reaction. To store the samples, 0.49 mg of 4-(2-aminoethyl) benzenesulfonyl fluoride (AEBSF) was added to the volume of the sample supernatant (200 µL) to inhibit further proteolytic degradation. The samples were shock-frozen at −80 °C to stop the degradation reaction as abruptly as possible.

### 2.7. Caco-2 Model

The Caco-2 model has several advantages: it is of human origin, has many characteristics analogous to the intestinal epithelium, and exhibits uptake properties consistent with in vivo observations. Caco-2 cells differentiate into a polarized monolayer exhibiting distinct apical and basolateral domains, tight junctions, and brush border characteristics similar to intestinal enterocytes [55,56]. They express key iron transport proteins including DMT1, ferroportin, and duodenal cytochrome b, enabling directional iron uptake and transport comparable to the human intestine [57,58,59,60]. Furthermore, Caco-2 cells regulate iron absorption in response to iron status and have been widely validated as a functional model of intestinal iron absorption [49,61]. Caco-2 cells (donation from the Molecular Gene- and Immuno Therapy Working Group, Charité-Universitätsmedizin Berlin, Germany) were grown in culture inserts in 24 well plates using Dulbecco’s Modified Eagle Medium (DMEM), which consisted of 10% FBS, 1% non-essential amino acids (NEAA), 1% Glutamaxx and sulfo-NHS-LC-Biotin (Biotin) (Thermo Fisher Scientific) 1% Antibiotic Antimycotic and 1% Pen strep. The medium was changed every 3 days. After 14–21 days, the cells began to form a monolayer, which separates the upper and lower surfaces of the cells, making it possible to study the permeability, absorption, and transport of substances across the epithelium. Transepithelial electrical resistance (TEER) measurements were carried out with Millicell ERS-2 (Merck KGaA, Darmstadt, Germany) to determine if the cells have a complete monolayer. The impedance should be >300 Ω·m^2^. The tight junctions of Caco-2 cells were examined using Alexa Fluor 488-labeled tight junction protein ZO-1 antibody. The cells were washed with PBS and fixed with 2.5% paraformaldehyde for 30 min at room temperature. After 30 min, permeabilization with 0.2% Triton X-100 was performed for another 5–10 min. The cells were then washed and blocked with 1% BSA for another 3 h. Finally, ZO-1 antibody (1:500) was added and the cells were incubated for another 2 h. After the time limit, the culture inserts were removed, the filters were removed, and the cells were examined under a confocal laser scanning microscope (CLSM) (LSM 510 META/Axiovert 200, Zeiss MicroImaging GmbH, Jena, Germany).

### 2.8. Viability of Caco-2 Cells

Metabolic activity/viability of Caco-2 cells: Cell-Counting-Kit 8 (CCK-8) was used to assess cell viability and cytotoxicity of the analyzed MPs. The CCK-8 test uses the water-soluble tetrazolium salt WST-8 [2-(2-methoxy-4-nitrophenyl)-3-(4-nitrophenyl)-5-(2,4-disulfophenyl)-2H-tetrazolium, monosodium salt] to induce a color reaction. In living cells, WST-8 is reduced by a dehydrogenase to an orange formazan dye, thus directly correlating with the number of living cells [62,63]. The cells were seeded into 96-well plates at a concentration of 5000 cells/well, diluted in 100 µL cell medium, and grown for 24 h. All incubations were carried out at 37 °C in the humidified air of a 5% CO_2_ incubator (HERACELL VIOS 160i; Thermo Scientific, Waltham, MA, USA). Afterward, the supernatants were removed and 100 µL of cell medium, containing IP-MPs from each particle type or iron solution (FLORADIX^®^ (Salus Pharma GmbH, Bruckmühl, Germany) and FeSO_4_) were added. FLORADIX^®^ (a commercially available iron substitute) as well as Fe SO_4_ are used as solutions with concentrations of 1.1, 2.8, and 11 µg/mL. Therefore, the iron concentration of the IP-MP suspensions was adjusted to the same values. For the control group, an equal volume of medium only without MPs was added to the cells. The cells were then incubated for 24 h, 48 h and 72 h, followed by the addition of 10 µL of a 50% CCK-8 solution in DPBS per well, and further incubation for 2 h. After incubation, the absorbance was measured at 450 nm using a microplate reader (PowerWave 340, BioTek Instruments GmbH, Bad Friedrichshall, Germany). The blank control group contained only medium and CCK-8 solution.

### 2.9. Iron Uptake of Caco-2 Cells

For the bioavailability experiments with the Caco-2 cells, the suspensions were used after passing through the 3-phase model. Caco-2 cells produce intracellular ferritin in response to increased iron uptake [49,64]. Measuring ferritin levels after exposure to iron serves as an indirect but sensitive indicator of iron absorption. Human ferritin ELISA Kit (Invitrogen, Carlsbad, CA, US) was used according to the manufacturer’s instructions. Briefly, an antibody specific for human ferritin was pre-coated onto the microplate wells. Ferritin in standards and samples is captured by the immobilized antibody. After washing, a biotin-conjugated detection antibody specific for human ferritin was added. Following another wash, Streptavidin–Horseradish Peroxidase (SA-HRP) was added, which binds to the biotin. After a final wash, a 3,3′,5,5′-Tetramethylbenzidine (TMB) substrate solution was added. The enzyme catalyzes a color change from clear to blue. The reaction was stopped with an acid solution, changing the color to yellow. The intensity of the yellow color was measured at 450 nm and was directly proportional to the concentration of ferritin in the sample.

## 3. Results and Discussion

The aim of the study was to determine whether the IP-MPs can reach the small intestine in the digestive model and be absorbed by the Caco-2 cells. In addition, it should be shown that the bioavailability of Fe(II) can be improved by the formulation of hybrid particles compared to conventional formulations to obtain new possibilities for iron supplementation in patients with anemia.

### 3.1. Particle Characterization

Iron–protein microparticles (IP-MPs) were fabricated using coprecipitation of iron sulfate and sodium carbonate to embed either BSA (IA-MPs) or hemp protein (IH-MPs). The properties of BSA and hemp protein differ in several aspects. The digestibility of BSA is approximately 95–99% compared with 85–90% for hemp protein; however, like BSA, hemp protein exhibits a high antioxidant capacity. This property could be advantageous for the protecting Fe(II) in IH-MPs and for applications in vegan diet.

**Figure 3:**
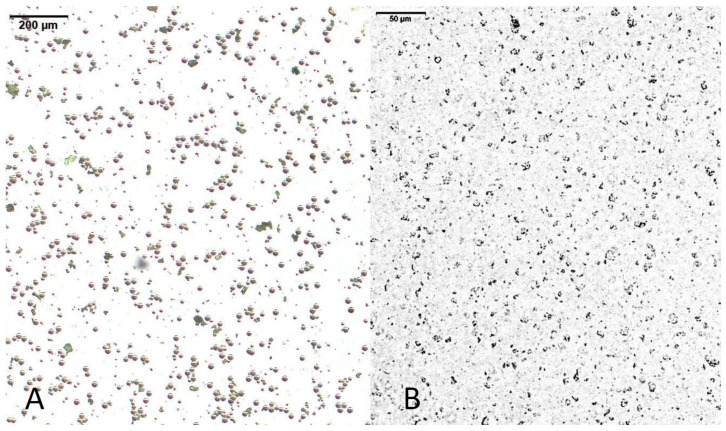
Microscopic images of (**A**) IA-MPs (scale bar: 200 µm); (**B**) IH-MPs (scale bar: 50 µm).

**Figure 4:**
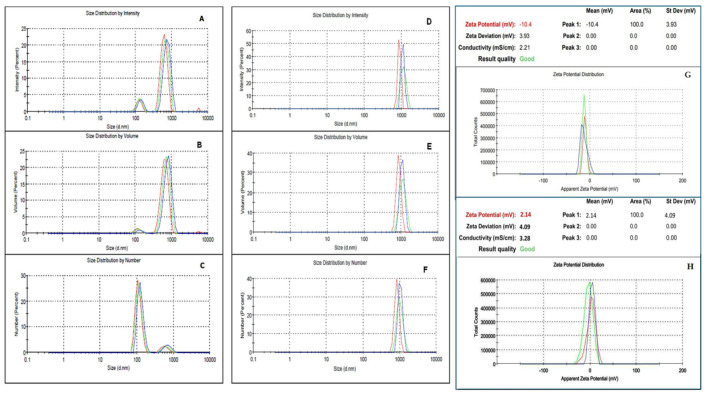
Size distributions of IA-MPs (**A**–**C**) and IH-MPs (**D**–**F**); zeta potential of IA-MPs (**G**) and IH-MPs (**H**). The colors of the curves represent three repeated measurements. The distributions differ depending on the weighting model used for particle size calculation. The IH-MP suspension is homogenous and shows no evidence of aggregation. The zeta potential of IA-MPs is negative, while that of IH-MPs is positive.

The overview of IA- and IH-MPs in Figure 3 shows that IA-MPs exhibit a more heterogenous size distribution compared with IH-MPs. This observation is further supported by the size distributions shown in Figure 4A–F. The IH-MPs exhibit particles predominantly around 1 µm, whereas IA-MPs show a broader distribution, including larger particles of approximately 800 nm and a substantial fraction around 100 nm.

Coprecipitation of proteins with iron salts is strongly influenced by protein structure, charge distribution, and metal-binding chemistry. In our experiments, albumin–iron sulfate systems produced a broad, heterogeneous particle size distribution, whereas hemp protein–iron sulfate coprecipitates exhibited a markedly more uniform particle size. Several mechanistic factors explain this contrast.

Albumin is a flexible, globular protein containing multiple high-affinity binding sites for both Fe^2+^ and Fe^3+^ ions. Metal binding induces partial unfolding, heterogeneous cross-linking, and multi-step aggregation processes, which generate particles with widely varying sizes and morphologies. The Zeta potential was −10.4 mV ± 3.9 mV (conductivity: 2.21 mS/cm). The low isoelectric point of albumin (pI ≈ 4.7) further contributes to heterogeneous charge neutralization during iron addition, resulting in uneven nucleation and growth of aggregates. These behaviors are consistent with earlier reports showing that serum albumins undergo conformational changes and irregular aggregation in the presence of transition metals [65,66].

In contrast, hemp protein isolates—dominated by the storage protein edestin—exhibit more rigid quaternary structures, fewer metal-binding motifs, and pI values closer to neutrality. The Zeta potential was 2.1 mV ± 4.1 mV (conductivity: 3.28 mS/cm). Under typical coprecipitation conditions, hemp proteins approach charge neutrality, enabling rapid, synchronous aggregation. This promotes the formation of more uniform nuclei and limits uncontrolled particle growth. Additionally, hemp protein isolates naturally contain polyphenols and small peptides that can act as metal-chelating and surface-active agents, modulating nucleation and stabilizing the growing particles. Similar effects of plant-derived phenolics and protein–metal interactions on controlled aggregation have been reported in other plant protein systems [67,68,69].

Differences in iron speciation may further amplify these effects. While albumin can bind mixed-valence Fe^2+^/Fe^3+^ species via strong chelation—resulting in heterogeneous cross-linking—hemp protein predominantly interacts through carboxylate-rich or phenolic sites in a more uniform fashion [70].

### 3.2. Digestion Model

**Figure 5:**
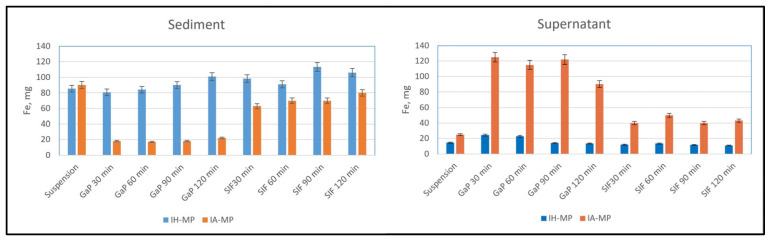
Stability (iron mass) of IH-MPs and IA-MPs in the different fluids of the digestion model: suspension (oral phase), GaP (gastric phase), and SIF (simulated intestinal fluid). Mean values ± SD (N = 3).

The stability of both albumin- and hemp-based MPs was investigated using an in vitro digestion model. Figure 5 shows the total iron content of IA-MPs and IH-MPs, measured in the sediment and supernatant, respectively.

Clear differences were observed between the stability of IH-MP and IA-MP suspensions. Due to the low pH in the gastric phase, IA-MPs were more readily degraded than IH-MPs. One likely reason is the inhomogeneous size distribution of IA-MPs, particularly the large proportion of particles smaller than 500 nm. In contrast, the larger and more stable IH-MPs were less affected by the acidic gastric conditions. Upon transition from the gastric to the intestinal phase, the pH increased, leading to an increase in IA-MP particle size. This effect is less pronounced in IH-MPs, indicating lower sensitivity to pH fluctuations.

**Figure 6:**
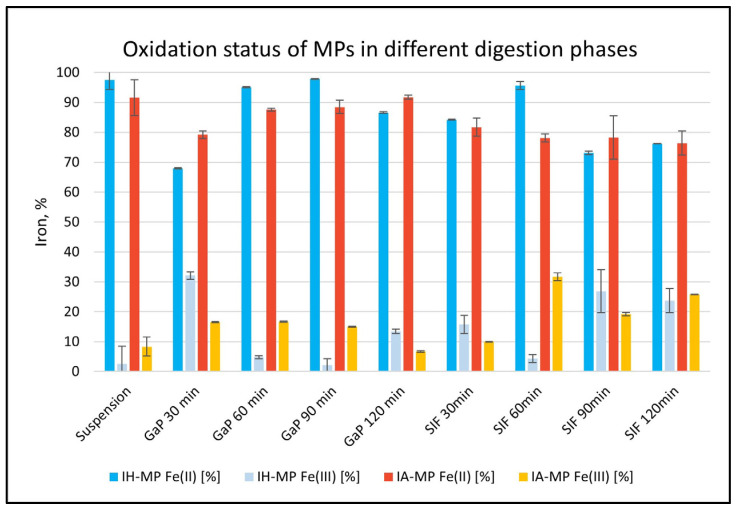
Percentage of Fe(II) and Fe(III) in IH- and IA-MP suspensions in different fluids of the digestion model: suspension (oral phase), GaP (gastric phase), and SIF (simulated intestinal fluid). The Fe(II) concentration in the MPs remains high throughout digestion for both particle types; mean values ± SD (N = 3).

During the transition of iron MPs from the gastric to the intestinal phase, an initial increase in Fe(III) was observed, followed by reduction back to Fe(II). A high Fe(II) concentration remained detectable throughout the entire digestion process in both IH- and IA-MPs (Figure 6). Many iron substitutes rely on stabilizing ligands (e.g., maltol, EDTA, polysaccharides); however, dietary and physiological inhibitors of iron absorption—such as phytates, polyphenols, calcium, and gastric pH—remain relevant obstacles for iron supplements and fortified foods [13]. Newer iron formulations (e.g., ferric complexes, polysaccharide-based, and liposomal iron preparations) aim to reduce side effects and improve tolerability; however, they still exhibit “lower bioavailability and slower uptake kinetics compared to ferrous salts” [71,72]. Even with more modern iron formulations (e.g., microencapsulated iron, and bis-glycinate), the clinical response—measured as reversal of iron deficiency—is not always superior, suggesting that absorption, which begins in the stomach and continues in the intestine, remains suboptimal in many substitutes [73,74]. Systemic regulation, particularly via hepcidin, can blunt iron uptake, meaning the mere presence of absorbable iron post gastric phase does not guarantee absorption [14,75].

### 3.3. Particle Uptake by Caco-2 Cells and Their Metabolic Activity

**Figure 7:**
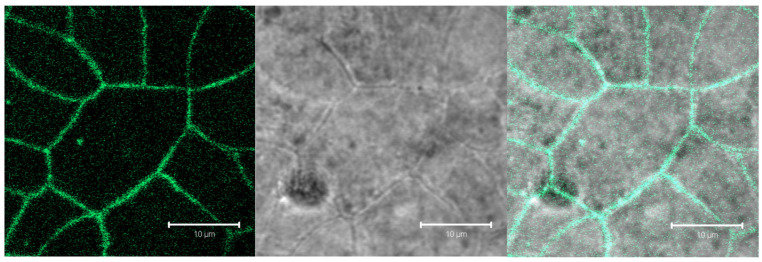
Confocal microscopic images of Caco-2 cells forming a closed monolayer. Tight junctions were labeled using an Alexa Fluor 488 antibody against the tight junction protein ZO-1. (**Left**): fluorescent image, (**Middle**): transmission image; (**Right**): overlay.

After demonstrating that the IP-MPs passed through the digestion model, we investigated whether IP-MPs could be taken up by Caco-2 cells as an in vitro model of the small intestine. Figure 7 shows the confluent monolayer of Caco-2 cells and the fluorescent labeled tight junctions. TEER (transepithelial electrical resistance) measurements were used to assess the integrity and tightness of the epithelial barrier by quantifying electrical resistance across the monolayer, thereby indicating the formation of functional tight junctions and barrier integrity.

**Figure 8:**
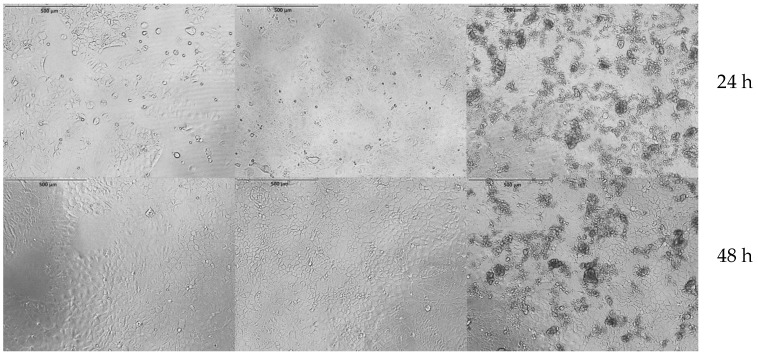
Caco-2 cells under control conditions (**left**), incubated with Floradix^®^ (**middle**), and incubated with IH-MPs (**right**). Upper row after 24 h; lower row after 48 h. No morphological changes were observed. Scale bar for all images, 500 µm.

**Figure 9:**
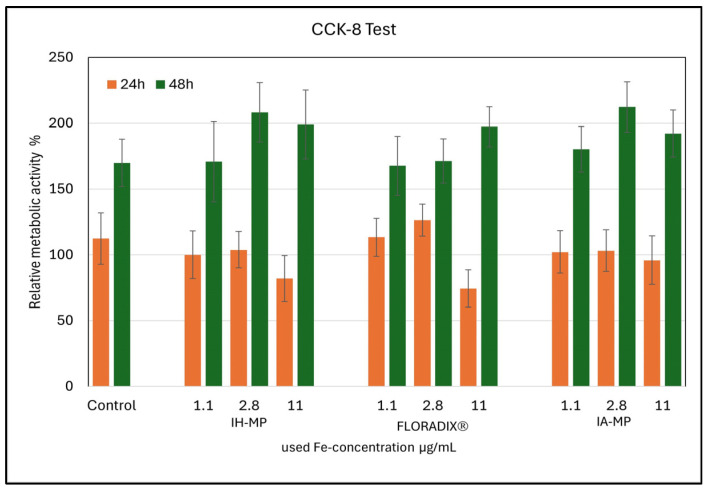
Relative metabolic activity of Caco-2 cells incubated for 24 and 48 h with IH-MPs, Floradix^®^, or IA-MPs. Iron concentrations of 1.1, 2.8, and 11 µg/mL. Mean values ± SD (N = 3).

After formation of closed Caco-2 cell monolayers, the different iron suspensions (Floradix^®^, IA-MPs, and IH-MPs) were added, and cell morphology, growth, and metabolic activity were analyzed (Figure 8). Metabolic activity, used as an indicator of cytotoxicity, was assessed using the CCK-8 assay after 24 and 48 h at three different iron concentrations (Figure 9).

After 24 h, metabolic activity measured by the CCK-8 assay was slightly reduced at higher iron concentrations for both Floradix^®^ and IH-MPs compared with control values or IA-MPs.

In contrast, after 48 h, all three iron formulations showed similar trends in metabolic activity. IH-MPs and IA-MPs exhibited higher metabolic activity compared with the control at iron concentrations of 2.8 and 11 µg/mL, respectively.

Several mechanisms may explain the increased “metabolic activity” observed in Caco-2 cells following iron exposure.

1. Iron is required for heme-containing proteins (complexes III–IV) and Fe–S clusters (complexes I–II and TCA-cycle enzymes such as aconitase). Increased bioavailable iron can enhance oxidative phosphorylation capacity and cellular dehydrogenase activity—the same biochemical processes responsible for reducing WST-8 (water-soluble tetrazolium-8) in the CCK-8 assay [76].
2. Iron loading can trigger transcriptional and metabolic remodeling, including shifts toward oxidative metabolism and antioxidant defenses, thereby increasing reducing equivalents detected by the CCK-8 assay [77].
3. Iron is indispensable for ribonucleotide reductase and numerous Fe–S enzymes involved in DNA metabolism. When iron is no longer limiting, cellular proliferation and biosynthesis increase, resulting in elevated WST-8 signals [78].
4. Labile Fe^2+^ can increase reactive oxygen species via Fenton chemistry; cells counteract this through NRF2-driven antioxidant responses and enhanced NAD(P)H generation, which can increase WST-8 reduction even at constant cell numbers [79].
5. Iron concentrations of 2.8 µg/mL (≈50 µM) and 11 µg/mL (≈197 µM) fall within ranges known to induce dose-dependent ferritin increases in Caco-2 cells [80].
6. IP-MPs contain proteins that, upon uptake by Caco-2 cells, may serve as additional nutrient or energy sources, thereby contributing to increased metabolic activity [81].

### 3.4. Transport of Iron Through the Caco-2 Cells

**Figure 10:**
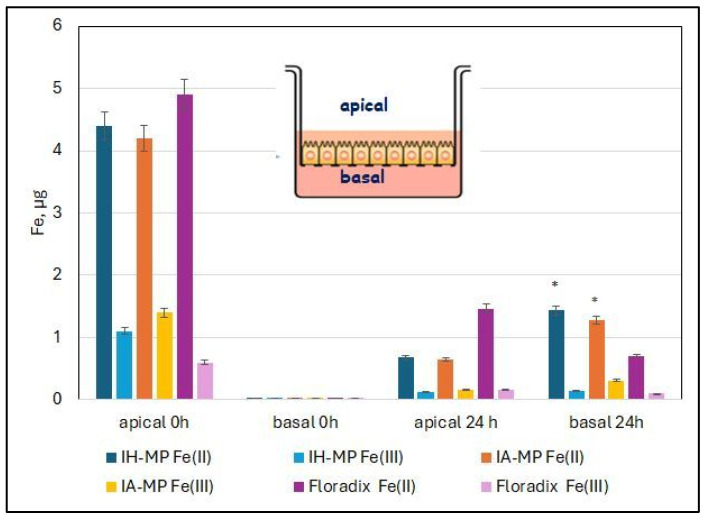
Transport of iron across the Caco-2 monolayer after 24 h. Fe(III) levels remained low for all preparations in both apical and basolateral compartments. The amount of Fe(II) in the basolateral compartment of IH-MP and IA-MP samples was significantly higher (*) than that of Floradix^®^. Mean values ± SD (N = 5, *p* < 0.05 Wilcoxon test).

The results of transport of iron across the Caco-2 cell layer are shown in Figure 10. A total of 5 µg of iron was applied for each preparation. Fe(III) levels remained low after 24 h for all preparations in both the apical and basolateral compartments. The amount of Fe(II) detected in the basolateral compartment of IH-MP and IA-MP samples (1.4 µg and 1.3 µg, respectively) was higher than that observed for Floradix^®^ samples (approximately 0.7 µg) and corresponded to about 25% of the total iron applied. After 24 h, more than half of the iron from all preparations remained within the cell layer or associated with the insert membrane.

Caco-2 cells took up more iron during the first four hours when iron was supplied in the form of iron–protein particles. This faster uptake can be attributed to endocytosis of the particles, as the iron is packaged in a solid particulate form. In contrast, iron in Floradix^®^ is present in dissolved form and is taken up by Caco-2 cells via pinocytosis or through specific membrane transporters.

### 3.5. Bioavailability of Iron

**Figure 11:**
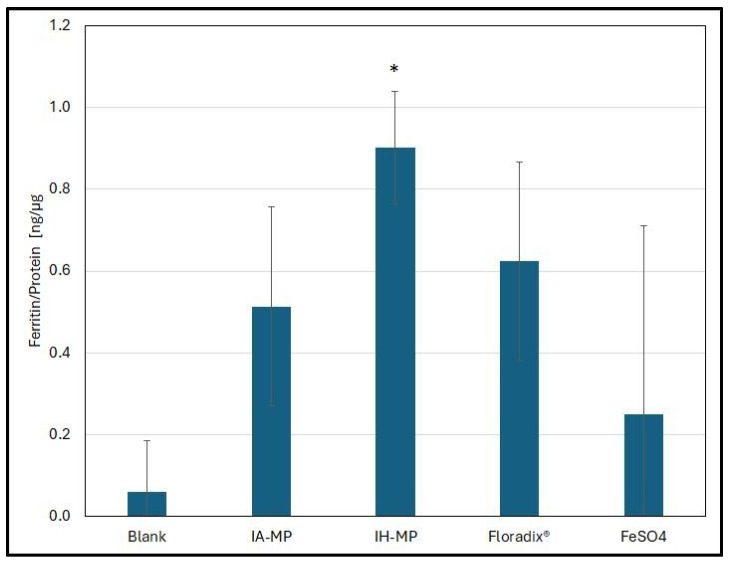
Ferritin-to-protein ratio in Caco-2 cells incubated with 5 µg of iron delivered as IP-MPs, Floradix^®^, or FeSO_4_. The ratio for IH-MPs was significantly higher (marked by *, *p* < 0.05, Wilcoxon test) compared with IA-MPs, Floradix^®^, and FeSO_4_, whereas no significant difference was observed between IA-MPs and Floradix^®^. Due to the high SD of the FeSO_4_ samples, no significant differences between FeSO_4_ and the other formulations could be confirmed. Mean values ± SD (N = 5).

After demonstrating that IP-MPs were taken up by Caco-2 cells, the remaining question was whether iron delivered via IP-MPs exhibited sufficient bioavailability. Figure 11 shows the ferritin content of Caco-2 cells following incubation with IP-MPs and for Floradix^®^ and FeSO_4_.

FeSO_4_ exhibited the lowest bioavailability. Floradix^®^ and IA-MPs induced significantly higher ferritin levels, although no significant difference was observed between the two formulations. IH-MPs yielded the highest ferritin response. In the standard in vitro digestion/Caco-2 assay, bioaccessible iron taken up by enterocyte-like Caco-2 cells induces ferritin synthesis. Because ferritin formation scales with the amount of iron entering the cells, ferritin content serves as the primary readout of iron bioavailability in this assay [64]. Contemporary applications compare iron sources and enhancers/inhibitors by the magnitude of ferritin induction after exposure to digested samples. This approach is explicitly described in updated protocols and recent studies using Caco-2 monolayers. A 2024 study reported that an iron–carnosine complex drove ~2.5× higher Caco-2 ferritin than FeSO_4_ at matched iron, and ~8× higher when co-present with ascorbate—illustrating how chemical form and enhancers translate into ferritin output [82]. Ferritin therefore reflects how much iron is taken up by enterocyte-like cells from the apical side. For this reason, ferritin is the principal outcome used to compare iron sources, chelates, and absorption enhancers or inhibitors. However, ferritin reflects cellular uptake rather than transepithelial export to the basolateral side, which is required for systemic absorption. This limitation has been noted in recent methodological overviews of Caco-2 systems; therefore, some studies combine Caco-2 cells with downstream cell models (e.g., HepG2) when basolateral export is of interest [83].

## 4. Conclusions

Our investigations were focused on the determination of the stability of IP-MPs in a static digestive model and their uptake by Caco-2 cells as an improved model of the intestinal epithelium and the bioavailability of Fe(II) using the formulation of hybrid particles compared to conventional formulations.

Albumin- and hemp protein-based iron microparticles provide an effective food-compatible strategy to enhance iron stability and cellular bioavailability. Those based on hemp protein are particularly promising candidates for food fortification and functional food applications aimed at improving iron nutrition. Especially considering the increasing problems with malnutrition in developing countries and ricing number of vegans and vegetarians in the industrial countries all over the world, IP-MPs could provide an effective food-compatible strategy with enhanced iron stability and cellular bioavailability.

Although the results are promising, it will be necessary to demonstrate in an anemic animal model that the iron–protein MPs can reduce anemia without significant side effects.

## Data Availability

The original contributions presented in this study are included in the article/Supplementary Material. Further inquiries can be directed to the corresponding author.

## Supplementary Materials

The following supporting information can be downloaded at: https://www.mdpi.com/article/10.3390/nu18071102/s1, File S1. Composition of SSF, SGF, and SIF/Preparation for 500 mL.
