# Supplementary material for: Bioavailability of Functional Iron in Protein Microparticles
Source: Nutrients. 2026 Mar 30;18(7):1102. doi: 10.3390/nu18071102 (PMC13074657; doi:10.3390/nu18071102)
Supplement: Supplementary file 1 [file nutrients-18-01102-s001.zip › nutrients-4209426-supplementary-File S1.pdf]

Composition of SSF, SGF, and SIF /Preparation for 500 mL

Simulated saliva fluid (SSF-stock solution)
- 15.1 mL of potassium chloride stock solution
- 3.7 mL of potassium dihydrogen phosphate stock solution
- 6.8 mL of sodium hydrogen phosphate stock solution
- 0.5 mL of magnesium chloride hexahydrate stock solution
- 0.06 mL of ammonium carbonate stock solution
- 0.09 mL of 1 M HCl for pH adjustment
- Sterile filtration (pore size 0.22 µm)

Simulated gastric fluid (SGF stock solution)
- 6.9 mL of potassium chloride stock solution
- 0.9 mL of potassium dihydrogen phosphate stock solution
- 12.5 mL of sodium hydrogen phosphate stock solution
- 11.8 mL of sodium chloride stock solution
- 0.4 mL of magnesium chloride hexahydrate stock solution
- 0.5 mL of ammonium carbonate stock solution
- 1.3 mL of 1 M hydrochloric acid for pH adjustment
- Sterile filtration (pore size 0.22 µm)

Simulated intestinal fluid (SIF-stock solution)
- 6.8 mL of potassium chloride stock solution
- 0.8 mL of potassium dihydrogen phosphate stock solution
- 42.5 mL of sodium hydrogen phosphate stock solution
- 9.6 mL of sodium chloride stock solution
- 1.1 mL of magnesium chloride hexahydrate stock solution
- 0.7 mL of 1 M hydrochloric acid for pH adjustment
- Sterile filtration (pore size 0.22 µm)

Stock solutions for the preparation of SSF, SGF, and SIF /Preparation of 1000 mL

Potassium chloride stock solution (0.5 M),
- 37.3 g potassium chloride
- Dilute to 1000 mL with deionized water
- Sterilely filter (pore size 0.22 µm)

Potassium dihydrogen phosphate stock solution (0.5 M),
- 68 g potassium dihydrogen phosphate
- Dilute to 1000 mL with deionized water
- Sterilely filter (pore size 0.22 µm)

Potassium dihydrogen phosphate stock solution (0.5 M),
- 68 g potassium dihydrogen phosphate
- Dilute to 1000 mL with deionized water
- Sterilely filter (pore size 0.22 µm)

Sodium chloride stock solution (2 M),
- 117 g sodium chloride
- Dilute to 1000 mL with deionized water
- Sterilely filter (Pore size 0.22 µm)

Sodium hydrogen phosphate stock solution (1 M),
- 84 g sodium bicarbonate
- Dilute to 1000 mL with deionized water
- Sterilely filter (pore size 0.22 µm)

Magnesium chloride hexahydrate stock solution (2 M),
- 30.5 g magnesium chloride hexahydrate
- Dilute to 1000 mL with deionized water
- Sterilely filter (pore size 0.22 µm)

Ammonium carbonate stock solution (0.5 M),
- 48 g ammonium carbonate
- Dilute to 1000 mL with deionized water
- Sterilely filter (pore size 0.22 µm)
